# Plasma Homocysteine Concentration is Associated with the Expression Level of Folate Receptor 3

**DOI:** 10.1038/s41598-020-67288-9

**Published:** 2020-06-24

**Authors:** Ren Yoshitomi, Kai Nakayama, Shuya Yamashita, Motofumi Kumazoe, Ting-An Lin, Chen-Yi Mei, Yuki Marugame, Yoshinori Fujimura, Mari Maeda-Yamamoto, Shinichi Kuriyama, Hirofumi Tachibana

**Affiliations:** 1 Division of Applied Biological Chemistry, Department of Bioscience and Biotechnology, Faculty of Agriculture, Kyushu University, Fukuoka, Japan; 2 Institute of Fruit Tree and Tea Science, National Agriculture and Food Research Organization, Makurazaki, Japan; 3 Food Research Institute, National Agriculture and Food Research Organization, Ibaraki, Japan; 4 Division of Molecular Epidemiology, Tohoku University Graduate School of Medicine, Sendai, Japan

**Keywords:** Cell biology, Biomarkers, Diseases, Health care, Molecular medicine

## Abstract

Folic acid and folate receptors (FOLRs) play an important role in the downregulation of homocysteine (Hcy), a risk factor of Alzheimer’s disease, thrombosis, neuropsychiatric illness and fractures. While several studies have reported that FOLR1 and FOLR2 import folic acid into cells, the role of FOLR3 remains unknown. In this study, we evaluated the impact of FOLR3 on the metabolism of Hcy alongside its protective effect against homocysteine-induced neurotoxicity. To reveal the role of FOLR3, we constructed FOLR3-overexpressed HEK293 cells (FOLR3^+^ cells) and evaluated cell growth, folic acid intake and Hcy-induced neurotoxicity. Subjects with a high expression of FOLR3 exhibited low levels of plasma homocysteine. The ectopic expression of FOLR3 enhanced cell growth, and the enhanced effect was neutralised by folic acid-deficient media. The Western blot analysis revealed that FOLR3 is secreted into cell supernatant. The folic acid intake of FOLR3^+^ cells was higher than that of wild-type cells. Supernatant from FOLR3^+^ cells showed a protective effect on Hcy-induced cytotoxicity. FOLR3 expression in plasma is negatively correlated with plasma homocysteine. Our study emphasizes the role of FOLR3 in the intake of folic acid into cells on the one hand and its protective role in Hcy-induced cytotoxicity on the other.

## Introduction

Homocysteine (Hcy) is a metabolite of cysteine and is produced from methionine when its terminal methyl group is removed. Hcy is recycled into methionine or converted into cysteine with folic acid^1^. High levels of Hcy in serum, above 15 µmol/L, create a medical condition known as hyperhomocysteinemia, which constitutes significant risk factors for several diseases, including Alzheimer’s disease, thrombosis, neuropsychiatric illness, cerebrovascular disease, dementia-type disorders and osteoporosis-associated fractures^2–5^. In the Japan Collaborative Cohort Study, a large population-based cohort study of middle-aged to elderly subjects on the lifestyle–disease relationship, revealed that people with a high serum homocysteine status (≧15.3 µmol/L) exhibit 4.4 and 3.4 times higher risk of ischaemic stroke and ischaemic heart disease, respectively^6^. Hcy is exacerbated by ageing, smoking and oxidative stress, which are known as risk factors for hyperhomocysteinemia^7,8^. Taken together, practical approaches to normalise Hcy levels are strongly recommended for persons who prone to developing hyperhomocysteinemia.

Folic acid is a water-soluble vitamin and is essential for hematinic processing and cell growth^9,10^. Tetrahydrofolic acid, a folic acid derivative, is produced from dihydrofolic acid by dihydrofolate reductase and plays a crucial role in acid and amino acid metabolism. Folic acid converts to 5-methyltetrahydrofolic acid in our body, which contributes to the remethylation of Hcy to methionine. Several epidemiological studies have revealed that folic acid deficiency can increase Hcy levels in the blood^11–13^.

The folic acid receptors (FOLRs) are involved in uptaking folic acid. FOLR has three isotypes in humans: FOLR1, FOLR2 and FOLR3. Gene homology exists for each receptor (FOLR1 and FOLR2, 68%; FOLR1 and FOLR3, 71%; FOLR2 and FOLR3, 79%), and folate-binding sites are probably conserved in all three receptor subtypes^14,15^. Glycosyl phosphatidylinositol -anchored FOLR1 and FOLR2 are widely localised in the cell membrane and transport folic acid into the cell by endocytosis. FOLR3 is a localised haematopoietic tissue, such as spleen and bone marrow, and is present as a secretory protein^16,17^. FOLR3 expresses in humans rather than mice and rats. Several studies have investigated the role of FOLR1 and FOLR2, whereas the role of FOLR3 remains unknown. This study aims to explore the relationship between FOLR3 and Hcy in our body; it also intends to investigate the impact of FOLR3 on folic acid metabolism.

## Results

### Plasma homocysteine concentration is negatively correlated with the expression levels of FOLR3 in plasma

**Figure 1:**
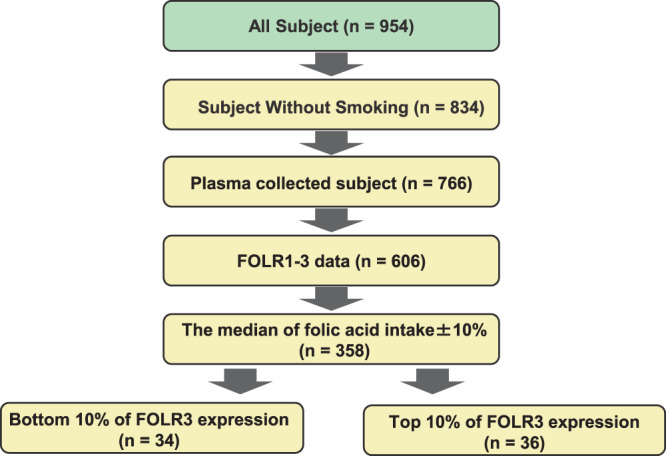
A flowchart of subjects included in this study.

**Figure 2:**
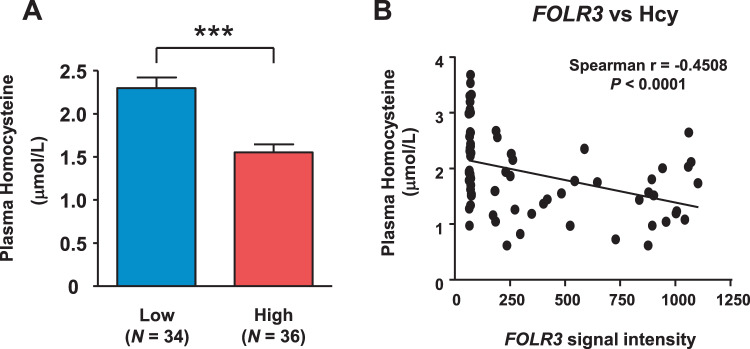
Plasma homocysteine concentration is negatively correlated with the expression levels of FOLR3 in plasma. (**A**) ELISA analysis of plasma homocysteine in subjects with the high or low expression levels of FOLR3; low expression level (n = 34) and high expression level (n = 36). (**B**) Spearman rank correlation analysis between plasma homocysteine and FOLR3 signal intensity.

Between the two groups (bottom 10% of FOLR3 expression and top 10% of FOLR3 expression), no significant difference in age, intake of folic acid, vitamin B6, vitamin B12 and expressions of FOLR1 and FOLR2 was found (Fig. 1). In contrast, FOLR3 expression in high FOLR3 group was 8.7 times higher than that in low FOLR3 group (Table 1). We observed a 33% decrease in plasma homocysteine concentration in the subjects with high FOLR3, compared with subjects with low FOLR3 (Fig. 2A). In addition, plasma homocysteine concentration is negatively correlated with the expression levels of FOLR3 in plasma (*r* = −0.45, *p* < 0.0001) (Fig. 2B). Moreover, FOLR3 showed a stronger negative correlation than FOLR1 and FOLR2 (Supplementary Fig. 1). These results suggest that FOLR3 is associated with the metabolism of plasma Hcy.

**Table 1:** Characteristics between the subjects of high level FOLR3 expression and low lwvwl FOLR3 expression.

	Low level of FOLR3	High level of FOLR3	P value
Age	52.6 ± 2.4	52.3 ± 2.0	0.92
The number of subjects (male ratio)	34 (23.5%)	36 (25.0%)	
Dietary folic acid intake (μg/day)	442.3 ± 5.0	448.9 ± 3.8	0.29
Dietary vitamin B_6_ intake (mg/day)	1.58 ± 0.02	1.60 ± 0.03	0.59
Dietary vitamin B_12_ intake (μg/day)	8.90 ± 0.22	8.97 ± 0.21	0.82
FOLR1 (signal intensity)	60.0 ± 10.3	78.1 ± 13.0	0.28
FOLR2 (signal intensity)	54.9 ± 9.42	72.5 ± 12.1	0.23
FOLR3 (signal intensity)	70.9 ± 12.2	610.8 ± 101.8	<0.0001

### FOLR3 and folic acid support cell growth

**Figure 3:**
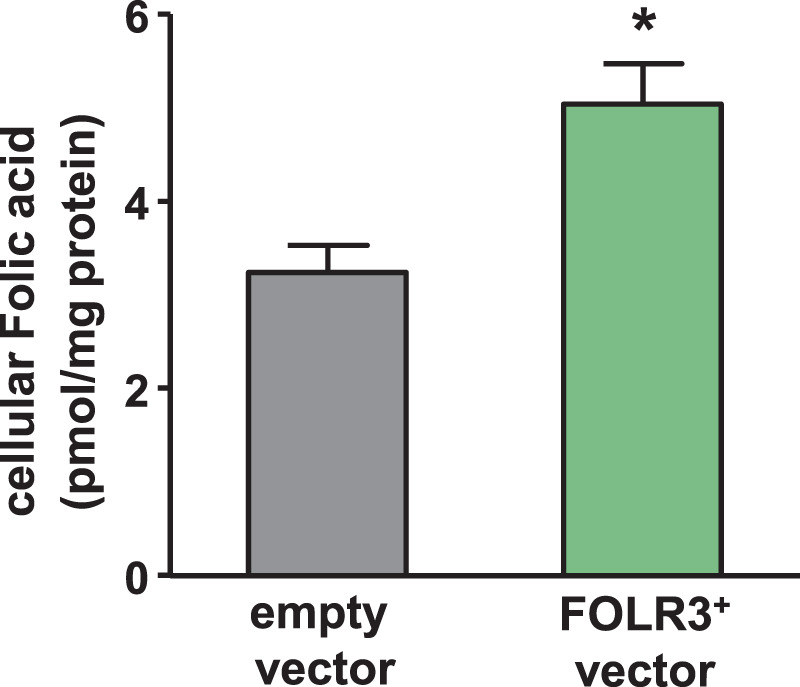
FOLR3 expression enhances folic acid uptake. Quantification of folic acid into cell with/without FOLR3. Unpaired t-test was used for statistical analysis and all data presented mean ± S.E. (n = 3). *p < 0.05, **p < 0.01 n.s.; not significant.

**Figure 4:**
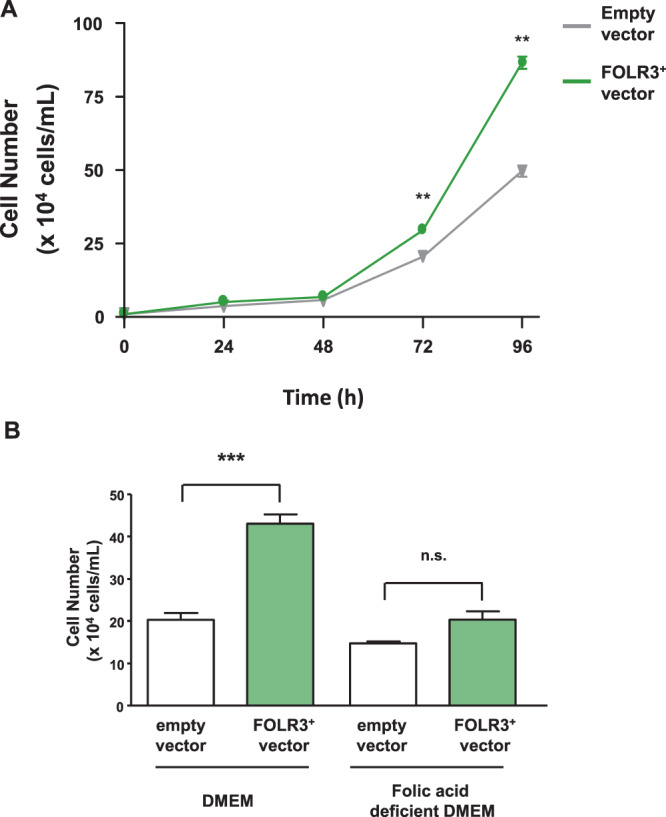
Involvement of FOLR3 and folic acid in cell growth of HEK293 cells. (**A**) The effect of FOLR3 expression on cell growth of HEK293 cells. (**B**) Cell growth of FOLR3-expressed HEK293 cells with/without folic acid. Two-way ANOVA with Sidak’s test for multiple comparisons was used for statistical analysis and all data presented mean ± S.E. (n = 3), **p < 0.01, ***p < 0.001 n.s.; not significant.

We hypothesised that FOLR3 enhances folic acid bioavailability and reduces plasma Hcy level. Thus, we constructed FOLR3-overexpressed HEK293 cells (FOLR3^+^ cells) (Supplementary Fig. 2). We used LC-MS to evaluate the folic acid concentration in the cells. The analysis results revealed that folic acid in FOLR3^+^ cells is 1.5 times higher than that in normal HEK293 cells (Fig. 3). Folic acid is vital for the growth and development of cells. Therefore, we monitored cell growth in HEK293 cells. The results revealed that cell growth is enhanced by the ectopic expression of FOLR3 (Fig. 4A). To assess whether the enhanced cell growth in FOLR3^+^ cells was associated with folic acid, FOLR3^+^ cells were cultured in DMEM with folic acid and then without it. Our results revealed that the number of FOLR3^+^ cells is higher than that of normal HEK293 cells in DMEM containing 4 mg/L folic acid, whereas no significant difference between FOLR3^+^ and HEK293 cells in DMEM without folic acid was discerned (Fig. 4B).

### FOLR3 is secreted into cell culture supernatant and reduces homocysteine-induced neurotoxicity

**Figure 5:**
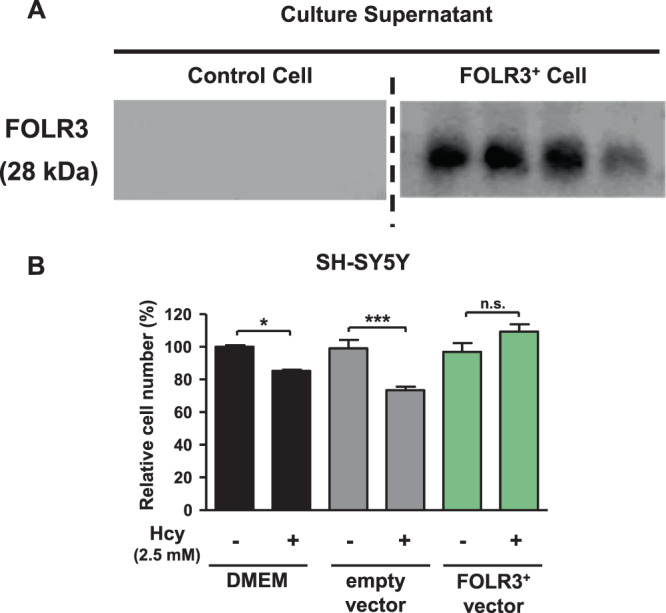
FOLR3 secretes into cell supernatant and reduces homocysteine induced-neurotoxicity. (**A**) The FOLR3 expression in the empty vector or FOLR3+ cells culture supernatant. (**B**) The effect of supetnatant of FOLR3-overexpressed HEK293 cell on homocysteine-induced toxicity in SH-SY%Y. Two-way ANOVA with SIdak’s test for multiple comparisons was used for statistical analysis and all data presented mean ± S.E. (n = 3) *p < 0.05, ***p < 0.001 n.s.; not signicant.

FOLR3 is a secretory and non-transmembrane protein. Thus, we assumed that the secreted FOLR3 would enhance folic acid bioavailability. The Western blotting analysis revealed that FOLR3 is secreted into cell supernatant (Fig. 5A). It is known that Hcy exerts neurotoxicity. We evaluated the effect of secreted FOLR3 on Hcy-induced neurotoxicity. Since the viable cells has been an indicator of neurotoxicity in other studies^18,19^, the number of viable cells was also used as an indicator of neurotoxicity in the present study. We discovered that supernatant from normal HEK293 cells did not decrease neurotoxicity whereas supernatant from FOLR3^+^ cells decreased Hcy-induced neurotoxicity (Fig. 5B). These data suggest that secreted FOLR3 mediate folic acid intake into cells and reduce Hcy-related toxicity.

## Discussion

Hyperhomocysteinemia refers to a condition, in which a high level of Hcy exists in the blood and is considered a risk factor for many diseases, such as Alzheimer’s disease, thrombosis, neuropsychiatric illness, stroke, dementia-type disorders and osteoporosis-associated fractures^2–5^. Folic acid plays a key role in reducing Hcy-related toxicity by recycling Hcy into methionine. Although folic acid reduces Hcy levels, randomised placebo-controlled clinical trials indicate that vitamin therapy, including folic acid therapy, has mixed impacts on Hcy-related diseases. It has been suggested that folic acid supplementation is not sufficient to ameliorate hyperhomocysteinemia^20–22^. Our study alludes to the potential effects of FOLR3 on Hcy-relating diseases.

FOLR3 is a secretory protein and is hypothesised to support the functions of folic acid and its derivatives. Nevertheless, it is difficult to evaluate the role of FOLR3 in animal experiments mainly because of the absence of orthologues in mice and rats. Our report is the first study, in which plasma FOLR3 is inversely correlated with plasma Hcy level; it is also the first study in which secreted FOLR3 mediates the folic acid intake of the interior cells and neutralises Hcy-induced toxicity. FOLR1 and FOLR2 transport folic acid and its derivatives to the cells and either recycle them into methionine or convert them into cysteine^23^. Gene homology exists for three FOLRs and folate-binding sites of three FOLRs are probably conserved^14,15^. Taken together, FOLR3 has the same impacts on folic acid functions as FOLR1 and FOLR2. However, the present study found that FOLR3 correlated more strongly with plasma Hcy than FOLR1 and FOLR2. Thus, FOLR3 may decrease plasma Hcy compared with other FOLRs. We also demonstrated that FOLR3 can metabolise both intracellular Hcy and extracellular Hcy. These results indicate that an increase in FOLR3 may effectively ameliorate Hcy in the blood and weaken Hcy-induced toxicity, even in tissues with the low level of FOLR1 and FOLR2 expression.

Some secretory proteins communicate with the organs and have several effects on our body. FOLR3 ameliorates Hcy cytotoxicity and is captured in SH-SY5Y cells. These results suggest that secreted FOLR3 may cause Hcy to have a minimal effect on the terminal tissue and reduce the Hcy level in plasma and the risk of Hcy-related diseases. The upregulation of FOLR3 and supplementation of folic acid can be advantageous treatment strategies for hyperhomocysteinemia patients and for the prevention of Hcy-related diseases.

P53 has the function of controlling the inhibition of apoptosis^24^. Hcy is known to induce p53 activation and DNA damage, exerting neurotoxicity^25^. In contrast, folic acid metabolizes homocysteine to methionine and induces p53 DNA methylation, supressing Hcy-induced toxicity^26^. Our study showed that FOLR3 upregulated folic acid concentration in the cells. These data indicate that secreted FOLR3 could induce p53 methylation by efficient folic acid uptake and affect the cell viability of SH-SY5Y cells.

Endocytosis involves the process of transporting molecules into the cell by engulfing it with its membrane. Folic acid internalises GPI-anchored FOLR1 and FOLR2 *via* endocytosis and exerts its functions in the cell. Thus, secreted FOLR3 is migrated to terminal tissues and may interact with endocytosis-related protein to induce endocytosis for the internalisation of folic acid.

Receptor expression affects the downstream of the receptor. For example, insulin receptor, upregulated by berberine, lowers the blood glucose level among patients with type 2 diabetes mellitus, whereas muscle-specific insulin receptor knockout mice elevate blood glucose level^27,28^. Enhancing FOLR3 expression may promote folic acid functions, such as the reduction of Hcy, production of nucleic acid and creation of red blood cells.

FOLR3 expression level may depend on the innate or adaptive response, such as single-nucleotide polymorphisms (SNPs), environmental factors, exercise and food intake. FOLR3 has seven types of SNPs, although functions of SNPs in the FOLR3 gene are still unknown^29^. Further research is necessary to investigate the correlation between FOLR3 and innate or adaptive response.

Several papers have been reported normal ranges of Hcy as about 4–15 μmol/L^30,31^. Plasma Hcy levels in our study is lower compared with those pervious study. This discrepancy may be due to the different measure approach and the storage time from the day the plasma was collected until the plasma homocysteine was measured.

## Material and Methods

### Kakegawa cohort study

The Kakegawa cohort study is previously described^32^. Human tissues used in this study were obtained in accordance with the Declaration of Helsinki, and informed consent was obtained from all the patients and healthy volunteers. We conducted a baseline survey among 1544 men and women who were aged over 30 years and who lived in Kakegawa City, Shizuoka Prefecture, Japan, from June 2009 to October 2011. The protocol of this study conformed to the relevant statutes and guidelines and was approved by the ethics committee of Tohoku University and Kyushu University. The recruitment and investigation methods in this research have been described previously^32^. In brief, we explained this research to the participants before distributing the research questionnaires.

### Participants’ characteristics

Previous studies have concluded that smoking increases plasma homocysteine; thus, this study excluded subjects who smoked. According to semiquantitative food frequency questionnaire, participants around the median of folic acid intake (452 μg/day) were extracted from the subjects with plasma and FOLR data. The extracted participants in the bottom or top 10% of FOLR3 expression are defined as ‘Low FOLR3’ and ‘High FOLR3’, respectively (see Fig. 1).

### Measurement of plasma Hcy concentration

plasma homocysteine concentration was measured using an Axis Homocysteine EIA Kit (Axis-Shield Diagnostics Ltd., Scotland, UK). In brief, the plasma volume used was 25 µl. And then, the absorbance was measured at 405 nm using an EnVision 2104 Multimode Plate Reader (PerkinElmer, Waltham, MA, USA).

### DNA chip assay

Following the manufacturer’s instructions, TRIzol reagent (Invitrogen, Carlsbad, CA, USA) was used to extract total RNA from the cells. The RNA quality was estimated using the Agilent RNA 6000 Nano Kit (Agilent, Santa Clara, CA, USA) with the Agilent 2100 Bioanalyzer. The RNA integrity number for all the samples was higher than 6.5 (on a 0–10 scale). MessageAm II aRNA Amplification Kit (Ambion, Austin, TX, USA) was used to synthesise antisense RNA (aRNA) from 1 mg of total RNA. 5 mg of aRNA was fragmented and hybridised in the prototype Food-Sensitivity-Evaluation DNA Chip, Genopal (Mitsubishi Rayon Co., Tokyo, Japan). The hybridised aRNA was labelled Cy5 (GE Healthcare Japan, Tokyo, Japan), and fluorescence was assessed using a detector system (Yokogawa Electronic Co., Tokyo, Japan). All gene expressions were normalised by the 8 reference genes [actin beta; ATP synthase subunit b, mitochondrial; glyceraldehyde-3-phosphate dehydrogenase; General Transcription Factor IIB; phospholipase A2 group V; ribosomal protein S5; Tubulin Alpha 1b and ribosomal protein, large, P0].

### Cell culture

Human embryonic kidney cell line HEK293 cells were cultured in 10% fetal bovine serum (FBS) with Dulbecco’s Modified Eagle’s Medium (DMEM) at 37 °C in a humidified atmosphere with 5% CO_2_. FOLR3 expression vector (pcDNA3.1+ /C-(K)-DYK [OriGene Technologies, Rockville, MD]) was transfected in HEK293. Human neuroblastoma SH-SY5Y cells were cultured in 10% FBS with a 1:1 mixture of Eagle’s Minimum Essential Medium and Ham’s F-12 Nutrient Mix at 37 °C in a humidified atmosphere with 5% CO_2_.

### Cell assay

To assess the role of FOLR3 in cell growth, HEK293 cells (containing empty vector) and FOLR3+ cells were pre-treated with 10% FBS-containing DMEM for 24 h, and then the media were exchanged with 10% FBS-containing DMEM. After incubation for 24 h, 48 h, 72 h and 96 h, auto blood cell counter (Sysmex, Hyogo, Japan) was used to count the cell number. To estimate the involvement of folic acid on cell growth of FOLR3+ cells, HEK293 cells (containing empty vector) and FOLR3+ cells were pre-treated with 10% FBS-containing DMEM for 24 h, and then the media were exchanged with DMEM or folic acid-depleted DMEM (containing 10% FBS). After incubation for 72 h, cultured cells were washed and calculated the same method as above. To test if secreted FOLR3 affects Hcy-induced toxicity, supernatant of HEK293 cells (containing empty vector) or FOLR3+ cells incubated with serum-free DMEM for 48 h were filtered by 0.2 μm filter. SH-SY5Y cells were pre-treated with 10% FBS-containing DMEM for 24 h, and then the media were exchanged with 2.5 mM Hcy and the supernatant of HEK293 cells (containing empty vector) or FOLR3+ cells. After incubation for 96 h, cultured cells were washed and calculated the same method as above.

### Western blotting

Western blot analysis performed as described previously^33^. In brief, cells were harvested in a cell lysis buffer. Approximately 50 mg of protein was diluted in a sample buffer. The samples were boiled and electrophoresed in SDS-polyacrylamide gels, and the gels were blotted to Trans-Blot nitrocellulose membranes (Bio-Rad Laboratories, Inc. Hercules, CA). The membranes were blocked with 2.5% BSA-TBST for 1 h, followed by overnight incubation with the following primary Abs diluted in 2.5% BSA-TBST: anti-Flag Ab and b-actin Ab. Membranes were rinsed three times with TBST and treated with an HRP-conjugated secondary Ab (anti-mouse or anti-rabbit IgG), diluted in 2.5% BSA-TBST. The labelled membranes were rinsed with TBST, and bands were detected using TMA-6 chemiluminescence agents (Lumigen, Southfield, MI), according to the manufacturer’s protocols. A Fusion System (Vilber Lourmat, Eberhardzell, Germany) was used to obtain the images.

### Quantitative analysis of folic acid

Cultured HEK293 cells were washed with ice-cold PBS, added 1000 μL of 5% ammonia and homogenised the cells with Handy Sonic model UR-20P (TOMY SEIKO, CO. LTD, Tokyo, Japan). Cell lysates were vacuum dried using Speed Vac SC110A (Savant Farmingdale, NY, USA). The residue was resuspended with 1: 1 acetonitrile: water (500 μL), and 20 μL of 100 μM MTX (solvent: acetonitrile: water = 1: 1 solution) was added as an internal standard. The cell lysate was vortexed for 20 seconds and then centrifuged at 12,000 xg for 10 minutes, and the supernatant was collected and dried using Speed Vac SC110A. The residue was reconstituted with 1: 1 acetonitrile: water (140 μL), vortexed for 20 seconds, centrifuged at 12,000 × g for 10 minutes, and 100 μL of the supernatant was collected as the sample. Samples (3 µL) were injected into 150 × 2.1 mm CAPCELL CORE C18 column (2.7 µm) (Osaka Soda, Osaka, Japan) performing LC-MS-8050 (Shimadzu Co., Kyoto, Japan). The mobile phase flow was 0.15 mL/min and operated at 40 °C. The mobile phase contained (A) 0.05% formic acid in H_2_O and (B) 0.05% formic acid in acetonitrile using a gradient elution of A–B (85:15) in 0–1 min, A–B (85:15) → A–B (25:75) in 1–5 min, A–B (25:75) → A–B (0:100) in 5–7 min, A–B (0:100) in 7–11 min, A–B (0:100) → A–B (85:15) in 11–11.5 min and A–B (85:15) in 11.5–15 min. The selected ion monitoring was used to operate the ESI in the positive ion mode. The folic acid (pteroylglutamic acid) concentration was normalised to the total protein concentration measured using the BCA method.

### Quantitative real-time analysis

Following the product’s instructions, TRIzol reagent (Thermo Fisher Scientific, MA, USA) was used to extract total RNA from cells and tissues. NanoDrop (Thermo Fisher Scientific, MA, USA) was used to assess the RNA concentrations based on absorbance at 260 and 280 nm. The quantitative real-time PCR-based analysis was conducted based on the relative standard curve method to quantify the expression of indicated genes. Briefly, 1.4 μL of cDNA was applied as the template for quantitative real-time PCR-based analysis based on the CFX manager according to the manual. The sequences for the primers using quantitative real-time PCR-based analysis were as follows: human actin beta (ACTB): forward; 5′-GGCACCCAGCACAATGAA-3′, reverse; 5′-CTAAGT. CATAGTCCGCCTAGAAGCA-3′. FOLR3: forward; 5′-GACGGACCTGCTCAATGTCT-3′, reverse; 5′-CGTGCAGCAGGCATTCTT-3′. The mRNA expression was calculated based on the cycle times of each gene. The cycle threshold of ACTB was performed for normalisation.

### Statistical analyses

All the statistical analyses included the unpaired *t*-test, two-way ANOVA with Sidak’s post hoc test or Spearman’s correlation tests using GraphPad Prism. Data are expressed as mean ± S.E. *P* > 0.05 is considered significant.

## Supplementary information

Supplementary information.
