# Supplementary material for: Plasma Homocysteine Concentration is Associated with the Expression Level of Folate Receptor 3
Source: Sci Rep. 2020 Jun 24;10:10283. doi: 10.1038/s41598-020-67288-9 (PMC7314855; doi:10.1038/s41598-020-67288-9)
Supplement: Supplementary file 1 — Supplementary information. [file 41598_2020_67288_MOESM1_ESM.pdf]

Plasma Homocysteine Concentration is Associated with the Expression Level of Folate Receptor 3

Ren Yoshitomi[1], Kai Nakayama[1], Shuya Yamashita[1,2], Motofumi Kumazoe[1], Ting-An Lin[1], Chen-Yi Mei[1], Yuki Marugame[1], Yoshinori Fujimura[1], Mari Maeda-Yamamoto[3], Shinichi Kuriyama[4] and Hirofumi Tachibana[1,*]

[1]Division of Applied Biological Chemistry, Department of Bioscience and Biotechnology, Faculty of Agriculture, Kyushu University, Fukuoka, Japan. [2] Institute of Fruit Tree and Tea Science, National Agriculture and Food Research Organization, Makurazaki, Japan. [3]Food Research Institute, National Agriculture and Food Research Organization, Ibaraki, Japan. [4]Division of Molecular Epidemiology, Tohoku University Graduate School of Medicine, Sendai, Japan *email: tatibana@agr.kyushu-u.ac.jp

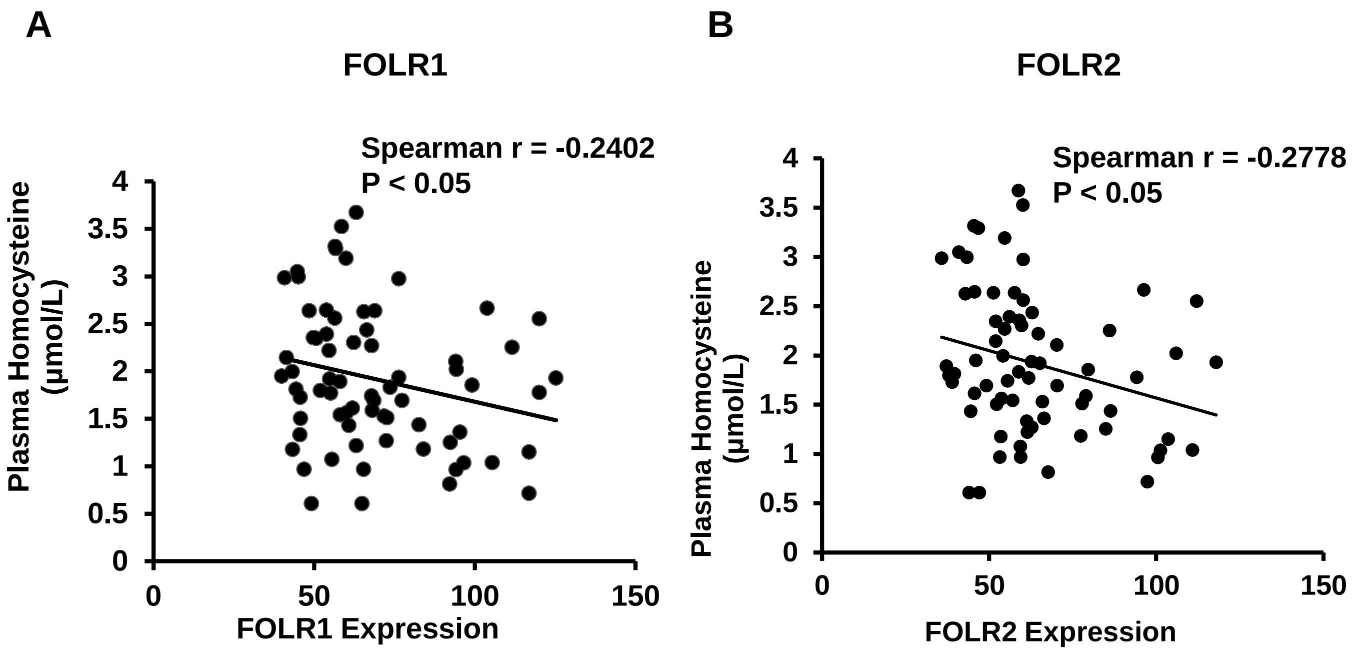

Supplementary figure 1. The correlation between plasma Hcy and FOLR1 or plasma Hcy and FOLR2. A),B）Spearman rank correlation analysis between plasma homocysteine and FOLR1 signal intensity (A) or FOLR2 signal intensity (B).

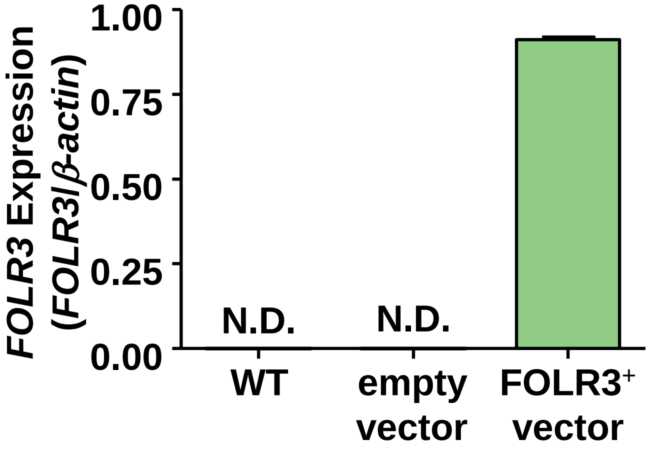

Supplementary figure 2. FOLR3 overexpression in HEK293.
data presented mean ± S.E. (n = 3) N.D.; not detected

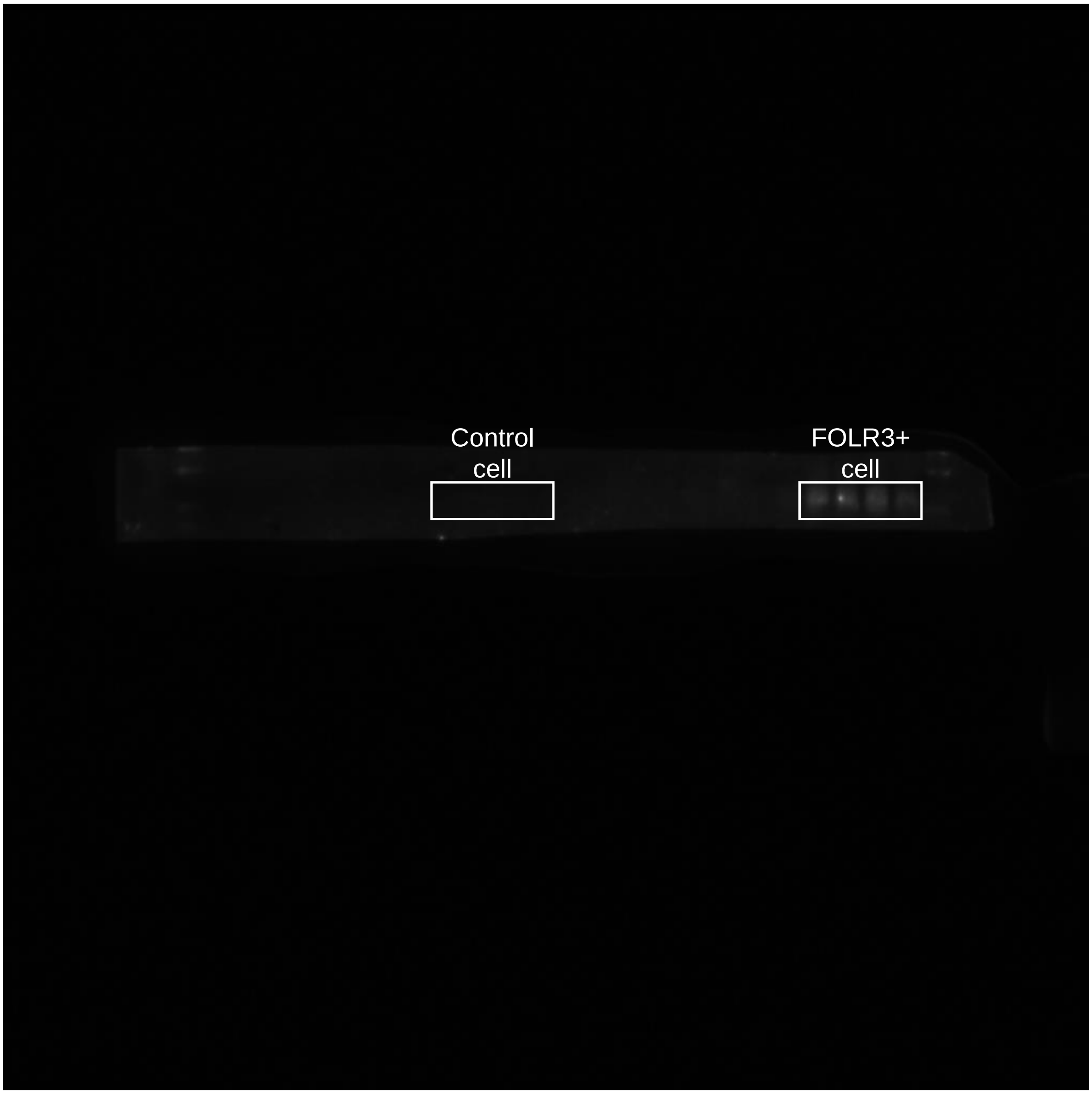

Supplementary figure 3. The raw data of figure 5A
